# Buffalo sperm surface proteome profiling reveals an intricate relationship between innate immunity and reproduction

**DOI:** 10.1186/s12864-021-07640-z

**Published:** 2021-06-26

**Authors:** Vipul Batra, Vanya Bhushan, Syed Azmal Ali, Parul Sarwalia, Ankit Pal, Seema Karanwal, Subhash Solanki, Arumugam Kumaresan, Rakesh Kumar, Tirtha Kumar Datta

**Affiliations:** 1 Animal Genomics Lab., Animal Biotechnology Centre, National Dairy Research Institute, Karnal, India; 2 Proteomics and Molecular Biology Lab, Animal Biotechnology Centre, National Dairy Research Institute, Karnal, India; 3 Theriogenology Lab, SRS of National Dairy Research Institute, Bengaluru, India

**Keywords:** Buffalo, Sperm, Beta-defensins, Epididymis, Glycosylation

## Abstract

**Background:**

Low conception rate (CR) despite insemination with morphologically normal spermatozoa is a common reproductive restraint that limits buffalo productivity. This accounts for a significant loss to the farmers and the dairy industry, especially in agriculture-based economies. The immune-related proteins on the sperm surface are known to regulate fertility by assisting the spermatozoa in their survival and performance in the female reproductive tract (FRT). Regardless of their importance, very few studies have specifically catalogued the buffalo sperm surface proteome. The study was designed to determine the identity of sperm surface proteins and to ascertain if the epididymal expressed beta-defensins (BDs), implicated in male fertility, are translated and applied onto buffalo sperm surface along with other immune-related proteins.

**Results:**

The raw mass spectra data searched against an *in-house* generated proteome database from UniProt using Comet search engine identified more than 300 proteins on the ejaculated buffalo sperm surface which were bound either by non-covalent (ionic) interactions or by a glycosylphosphatidylinositol (GPI) anchor. The singular enrichment analysis (SEA) revealed that most of these proteins were extracellular with varied binding activities and were involved in either immune or reproductive processes. Flow cytometry using six FITC-labelled lectins confirmed the prediction of glycosylation of these proteins. Several beta-defensins (BDs), the anti-microbial peptides including the BuBD-129 and 126 were also identified amongst other buffalo sperm surface proteins. The presence of these proteins was subsequently confirmed by RT-qPCR, immunofluorescence and in vitro fertilization (IVF) experiments.

**Conclusions:**

The surface of the buffalo spermatozoa is heavily glycosylated because of the epididymal secreted (glyco) proteins like BDs and the GPI-anchored proteins (GPI-APs). The glycosylation pattern of buffalo sperm-surface, however, could be perturbed in the presence of elevated salt concentration or incubation with PI-PLC. The identification of numerous BDs on the sperm surface strengthens our hypothesis that the buffalo BDs (BuBDs) assist the spermatozoa either in their survival or in performance in the FRT. Our results suggest that BuBD-129 is a sperm-surface BD that could have a role in buffalo sperm function. Further studies elucidating its exact physiological function are required to better understand its role in the regulation of male fertility.

**Supplementary Information:**

The online version contains supplementary material available at 10.1186/s12864-021-07640-z.

## Introduction

The voyage of the spermatozoa in the female reproductive tract (FRT) entails surmounting of numerous impediments including the physical, thermal, chemical and immunological barriers. These include the vaginal acidic pH, the mucus in the cervix, the leukocytes and anti-sperm antibodies of the immune system especially in the uterus, and the narrow utero-tubal junction in the oviduct [1–5]. To overcome these obstructions the spermatozoa must acquire surface properties primarily customized for this arduous journey. The process of sperm surface remodelling (SSR), which occurs during the epididymal transit of the spermatozoa tailors the sperm surface which assists them in survival and fertilization in the FRT [6–8]. The bio-molecular constitution of the mammalian testicular spermatozoa changes continuously and progressively in the luminal fluid of the various epididymal regions due to the secretory and re-absorptive actions of the epithelial cells that line this organ [9–12]. The remodelling events include a) enzymatic cleavage of the membrane-associated proteins b) variations in the composition of membrane-lipids c) re-organization of the glycoconjugates (GCs) associated with the sperm glycocalyx d) removal or addition of (glyco) proteins [9, 13, 14]. A blend of distinct secretagogues is known to be added onto the sperm-surface in these three epididymal regions viz. caput, corpus and cauda. A majority of these secretagogues include the immune-related (glyco) proteins often implicated in sperm survival and fertility. The epididymal secreted proteins which are involved in sperm maturation could be loosely adhered to the sperm plasma membrane or could be transmembrane. Many of these proteins bind transiently, for example, the ones acquired in the distal epididymal regions [15, 16]. These loosely adhered proteins in the peripheral sperm environment bind the sperm-surface either by electrostatic or hydrophobic interactions. These proteins change the sperm-surface characteristics as they interact with the transiting spermatozoa. The epididymal secretome also involves the glycan-modifying enzymes such as glycosidases and glycosyltransferases [17–19], proteases and protease inhibitors [10, 11, 20], proteins involved in immunological protection [3, 4] and the ones that protect the sperm from oxidative injuries [18, 21]. Besides, various membranous, extracellular vesicles (EVs) rich in cholesterol, sphingolipids and Ca^+ 2^ known as epididymosomes have been reported to exist in the epididymal lumen [22, 23]. These EVs mainly carry the GPI-APs, many of which are inserted in the sperm plasma membrane [24]. As mentioned earlier, many of the added (glyco) proteins on sperm-surface belong to defence family and their glycosylation patterns are critical for either stabilizing the sperm-membrane during the immune attack by immune cells or assisting in immune-evasion in the FRT [25–28]. The rendering of a highly glycosylated surface coat on the spermatozoa, not only acts as a barrier between the spermatozoa and female immune system but also assists spermatozoa in cervical mucus penetration (CMP), oviductal epithelial cell (OEC) binding, identification of the zona pellucida and oolemma, apposition of the sperm and the oocyte plasma membrane [3, 19, 29–31]. The sperm-surface proteins and their associated glycans also play a key role in the acquisition of motility and fertilizing ability in the epididymis, their protection, selection and secondary maturation in the FRT [19, 32].

The buffalo was considered as a model for this study due to its economic importance in agriculture-based economies. It has been reported that more people depend on buffalo than on any other domestic animal [33]. Although it is a premier dairy animal with superior milk-producing ability, idiopathic male infertility is a common reproductive limitation in buffalo. A sizeable number of high genetic merit bull calves originally selected for AI programs are discarded because their semen ends up yielding dismal conception rates (CRs) between 30 to 50%, reflecting poor fertilizing ability [34–36]. The factors that contribute to male fertility are relatively poorly understood, especially in bovine species [37]. The prediction of fertility assessment currently relies on analyses of sperm functional parameters apart from the physical examination of the bulls, nonetheless, the correlation between these parameters and the CR is often inconsistent [38]. Therefore, a better understanding of the novel factors which regulate fertility e.g. sperm-surface proteins is required to gain insights into the factors behind idiopathic male infertility.

The objective of this study was the identification and in silico characterization of the post-testicular maturation antigens and peripheral proteins that interact with the buffalo sperm plasma membrane either through non-covalent (ionic) interactions or through a GPI-anchor. We also sought to determine the existence of the epididymal expressed BDs on buffalo spermatozoa and to predict their reproductive functional significance through RT-qPCR, immunofluorescence and in vitro fertilization experiments.

## Results

The ejaculates that were milky or creamy in colour, homogenous in consistency i.e. free from flakes/clumps with a minimum sperm concentration of 600 × 10^6^/ml were considered for swim-up and further downstream experiments. The average motility and viability of seven representative sample ejaculates after processing was 81.84 ± 1.20% and 85.85 ± 1.16%, respectively. The samples were diluted according to the experiments, as mentioned, wherever required.

### Hundreds of proteins could be extracted from the buffalo sperm-surface after elevated salt (DPBS) or PI-PLC treatment

The extracted sperm-surface proteins identified after LC-MS/MS data processing indicated enough diversity among the types of proteins removed using the two treatment classes representing the a) the elevated salt extractions (2x-30, 2x-60, 4x-30, 4x-60) and b) the PI-PLC extractions (1 U/mL, 1.5 U/mL and 2 U/mL) (Supplementary Fig. 1 and 2). The extracted sperm-surface proteins produced > 20,000 PEP-XML spectra for each of the sub-groups of either treatment (elevated salt or PI-PLC). The iprophet tool correctly identified more than 300 proteins in all of the treatments at *p* > =0.99 where p indicates the probability that the spectra have been correctly matched to its analogous peptide (Table 1). A total of 317, 391, 394 and 432 proteins were identified in 2x-30, 4x-30, 2x-60 and 4x-60 (DPBS) treatments respectively. On the other hand, 385, 353 and 364 proteins were identified in the 1 U, 1.5 U and 2 U/mL PI-PLC treatments, respectively. At *p* ≥ 0.99 (iProphet probability) zero proteins were found to be incorrectly identified. Many proteins were found to be unique to each sub-group of either treatment demonstrating that the individual combinations of incubation time and salt/enzyme concentration exerted disparate effects on disrupting the non-covalent/GPI mediated interactions of the buffalo sperm surface proteins. Moreover, nearly 30% of the proteins were common between any two treatment subgroups (Supplementary Fig. 1 and 2).

**Table 1:** The seven treatment sub-groups for sperm-surface protein extraction from two treatment classes and the corresponding TPP results indicating total spectra, correctly and incorrectly identified proteins at *p* ≥ 0.6 and 0.99

Sample	PEP-XML Total spectra	Ipro.pep.xml Total spectra	Prot.xml Total proteins	Total proteins with ***p*** ≥ 0.6	Incorrectly identified with iProphet ***p*** ≥ 0.6	Total proteins with ***p*** ≥ 0.99	Incorrectly identified with iProphet ***p*** ≥ 0.99
2x-DPBS-30 min	26,535	7252	1733	875	76	317	0
4x-DPBS-30 min	26,470	8399	1725	956	76	391	0
2x-DPBS-60 min	25,947	7742	1898	1013	90	395	0
4x-DPBS-60 min	25,951	8210	2162	1244	99	432	0
1 U/ml	24,829	6557	1788	992	99	385	0
1.5 U/ml	24,587	6343	1932	1005	76	353	0
2 U/ml	24,881	5978	1662	793	63	364	0

Overall, we report a total of 352 buffalo sperm-surface proteins that were identified in the protein fractions extracted by the two treatment classes. The LC-MS/MS data analysis identified several BDs including the two Class-A beta-defensins (CA-BDs) viz. BD-129 and 126 amongst the other sperm-surface proteins Notwithstanding, only 85 proteins were found to be common among the elevated salt and PI-PLC treatments which were predicted to be extracellular or present on the buffalo sperm surface (Supplementary Fig. 2 and Supplementary sheet-Results). A remarkable diversity in the range of MW and pI was observed among these proteins (Supplementary sheet-Results). Amongst the mapped entries, the BDs like BD-134, BD-126 and Spag-11D were found to be among the proteins with the lowest molecular weight (M_r_ = 5.33, 7.44 and 11.95, respectively) while the angiotensin-converting enzyme (ACE) and the two uncharacterized proteins (UniProt ID: F1MD73 and F1MQ37) were the on the other end of the scale (M_r_ = 141.24, 190.10 and 227.10 respectively) (Supplementary sheet-Results-Mapped Entries). The BDs like Spag-11D, BD-129 and BD-126, however, had high pI values (9.5, 9.49, and 9.48, respectively) whereas the Acrosin inhibitor 1 had the lowest pI (4.25) among the mapped entries. Only three proteins viz. Sperm acrosome membrane-associated protein1, Angiotensin-converting enzyme and an uncharacterized protein (F1MD73) were predicted to contain a transmembrane segment (Supplementary sheet-Results-Mapped Entries). A high level of PTMs, especially glycosylation appears to modify the analyzed proteins because more than 80% of the analyzed proteins were predicted to possess at least either 1 N-glycosylation site or one O-glycosylation site. BD-126 was predicted to contain two O-glycosylation sites whereas the BD-129 predicted to contain eight such sites (Supplementary sheet-Results). The BD-126 and 134 were predicted to contain 1 N-glycosylation site while the BD-129 was predicted to contain three such sites.

### Proteins involved in the immune response and reproductive processes adorn the buffalo sperm surface

**Fig. 1:**
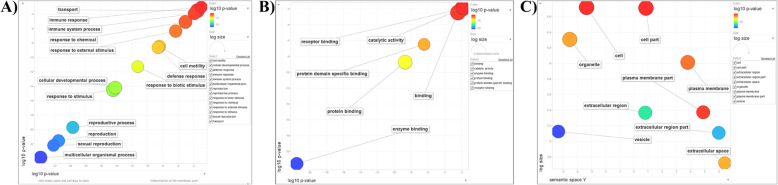
The semantic-similarity based scatter plots depicting the summarized lists of GO terms for **a** Biological Process, **b** Molecular Function and **c** Cellular Component domains for the buffalo sperm surface proteins

The gene ontology (GO) analysis was performed on the 85 extracellular (EC) sperm-surface proteins which were common between the two treatment classes (Supplementary Fig. 2c) wherein the annotation terms for Biological Process, Molecular Function and Cellular Component were determined (Fig. 1 and Table 2). These proteins were successfully mapped to 63 entries in the background dataset. The singular enrichment analysis (SEA) for Biological Process terms identified reproductive processes, sexual reproduction, immune response and response to biotic/abiotic stimulus terms as the major GO annotations in the input list *vis-à-vis* the background reference dataset, the Bovine genome locus (Bovine Genome Database): GLEAN_03528 (Table 2). The scatter plot analysis (SPA) for Biological Process similarly indicated higher semantic similarities between reproductive process functions, immune response and response to biotic/abiotic stimulus terms as observed by their closeness in the displayed two-dimensional space (Fig. 1a). The SEA for Molecular Function indicated that the majority of proteins were involved in catalytic and binding (carbohydrate or protein) functions (Table 2). The SPA for Molecular Function also identified protein binding and catalytic activity as the major GO terms with the highest uniqueness index values and the least dispensability scores (Fig. 1b and Supplementary sheet-Results). Most of the proteins were found to be extracellular, vesicular or part of the plasma membrane as indicated by the SEA and SPA for the Cellular Component terms (Fig. 1c and Supplementary sheet-Results). The low *p*-values from the Fisher’s test and the results of the Yekutieli test (low FDRs) are indicative of high confidence in the determined annotation terms for the input list in the SEA (Table 2). Similarly, the lower log_10_ *p*-values and dispensability score with high uniqueness index indicate the reliability of the GO annotation terms for the input list used for the SPA (Supplementary sheet-Results). Overall, these results suggested that the buffalo sperm surface is adorned with extracellular/vesicular-origin proteins which are involved in reproduction specific activities, immune responses, responses to biotic/abiotic stimuli usually performing catalytic or carbohydrate/protein binding functions.

**Table 2:** The major GO annotation terms, Fisher’s *p*-values and Yekutieli result (FDR under dependency) for the singular enrichment analysis (SEA) of Biological Process, Molecular Function and Cellular Component annotation terms performed on the sperm-surface proteins in the input list

Biological Process			Molecular Function			Cellular Component		
Term	***p***-value	FDR	Term	***p***-value	FDR	Term	***p***-value	FDR
Multicellular organismal process	2.00E-27	1.40E-24	Enzyme binding	2.90E-24	2.90E-22	Membrane-bounded vesicle	5.70E-85	6.10E-83
Sexual reproduction	1.90E-25	6.60E-23	Ubiquitin protein ligase binding	8.20E-16	4.10E-14	Vesicle	5.70E-85	6.10E-83
Reproduction	1.30E-24	3.00E-22	Protein binding	6.00E-11	2.00E-09	Extracellular region part	3.80E-70	2.70E-68
Reproductive process	1.20E-22	2.10E-20	Unfolded protein binding	3.60E-09	9.00E-08	Extracellular region	3.60E-62	1.90E-60
Positive regulation of biological process	1.90E-21	2.70E-19	Protein domain specific binding	1.80E-08	3.50E-07	Cytoplasm	3.90E-32	1.60E-30
Anatomical structure development	4.70E-21	5.50E-19	Carbohydrate binding	8.40E-06	0.00014	Membrane-bounded organelle	1.60E-29	5.70E-28
Regulation of biological quality	4.20E-18	4.20E-16	Binding	0.00037	0.0037	Extracellular space	9.30E-25	2.20E-23
Positive regulation of cellular process	5.30E-18	4.70E-16	Nucleotide binding	0.00041	0.0037	Organelle	1.60E-23	3.50E-22
System development	1.10E-17	8.90E-16	Enzyme regulator activity	0.00041	0.0037	Intracellular part	8.60E-20	1.70E-18
Response to stimulus	5.10E-17	3.60E-15	Catalytic activity	0.0022	0.012	Plasma membrane	7.80E-18	1.30E-16
Cellular developmental process	1.10E-16	7.00E-15	Receptor binding	0.0024	0.012	Cytoplasmic membrane-bounded vesicle	4.80E-15	6.10E-14
Response to biotic stimulus	1.90E-13	5.60E-12				Plasma membrane part	5.00E-09	3.90E-08
						Cell part	4.80E-06	2.60E-05
						Cell	4.80E-06	2.60E-05

### Cytometry reveals removal of glycosylated proteins from buffalo sperm surface after elevated salt/PI-PLC treatment

**Fig. 2:**
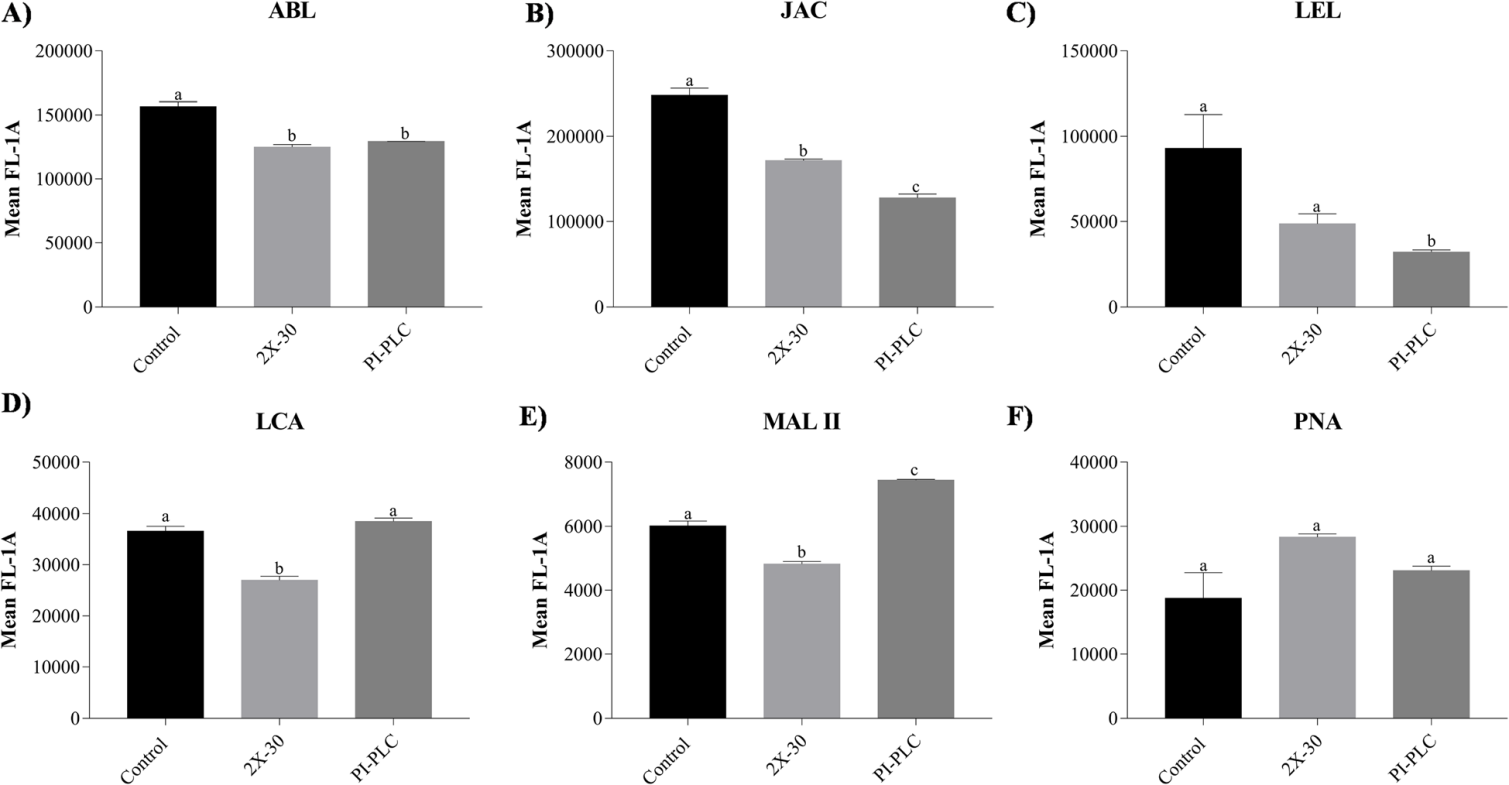
Histogram plots of the observed mean fluorescent intensity (MFI) values produced upon binding of six different FITC-labelled lectins viz. **a** ABL, **b** JAC, **c** LEL, **d** LCA, **e** MAL-II, and **f** PNA on buffalo bull spermatozoa in NCM (control), 2x-DPBS (2x-30) or spermatozoa exposed to 2 U/mL PI-PLC. The differences being assessed by one way ANOVA followed by Tukey’s multiple comparison test

The Flow cytometry analyses were performed on the control spermatozoa sample (NCM), spermatozoa incubated in elevated salt for 30 min (2x-DPBS) and spermatozoa incubated with PI-PLC (2 U/mL) to assess the corresponding changes in sperm-surface glycosylation after either treatment. The analyses revealed a reduction in both the O-linked and the N-linked glycans after either treatment as assessed by the decline in the MFI values which are produced upon binding of FITC-bound lectins on the buffalo sperm-surface (Fig. 2). A panel of five O-linked glycans specific lectins viz. ABL, JAC, MAL-II, LCA, PNA and 1 N-linked glycan specific lectin, LEL was used. The Brown-Forsythe test for all the lectins was found to produce a non-significant *p*-value (*p* > 0.05) indicating no differences in standard deviations of the MFIs produced in these groups. The unstained spermatozoa were excluded from the analysis by gating and the singlets were chosen for analyses which were performed on single, stained spermatozoa. The O-linked glycan-binding lectin, ABL preferentially binds the Thomsen-Friedenreich antigen, galactosyl (β-1, 3) N-acetylgalactosamine [39]. It produced a mean fluorescence intensity (MFI) of 1, 56,610.0 units in the control sample which differed significantly (*p* < 0.001) from the MFI produced in the spermatozoa incubated in 2x-DPBS (1, 25,032.0 units) or treated with PI-PLC (1, 29,399.0 units) as assessed by one-way ANOVA (Fig. 2 and Supplementary Fig. 3). The lectin JAC which has a sugar specificity towards galactose of O-linked glycans preferring the structure galactosyl (β-1, 3) N-acetylgalactosamine also produced higher MFI in the control sample (2, 47,848.0 units) in comparison to either the 2x-30 sample (1, 71,757.0 units) or the PI-PLC treated sample (1, 27,951.0 units). The post-hoc analysis indicated that the reduction in MFI for JAC after either the salt treatment (*p* < 0.001) or the PI-PLC treatment (*p* < 0.0001) was not only significantly different from the control sample but also each other (*p* < 0.01) (Fig. 2 and Supplementary Fig. 3). The N-linked glycan-binding lectin LEL which is specific for [GlcNAc] 1–3, N-acetylglucosamine is also removed from the sperm surface on exposing the spermatozoa to elevated salt milieu producing a diminished MFI of 48,715.0 units which didn’t differ significantly from the MFI produced in control samples (92,968.0 units) (Fig. 2 and Supplementary Fig. 3). The exposure of PI-PLC, nonetheless, reduced the MFI fluorescence significantly to 32,161.0 units (*p* < 0.05). The LCA lectin which is specific for mannose and glucose produced significantly (*p* < 0.001) reduced MFI of 26,979.0 units in the elevated salt-treated spermatozoa when compared to the control sample (36,559.0 units) (Fig. 2 and Supplementary Fig. 3). Conversely, the MFI increased minutely to 38,451.0 units after exposure to PI-PLC. The α-2, 3 linked sialic acid-binding lectin MAL-II (*p* < 0.001) similarly produced a significantly higher MFI in the control sample (6015.0 units) in comparison to the 2x-30 sample (4820.0 units) (Fig. 2 and Supplementary Fig. 3). Nevertheless, as observed for LCA binding, the MFI increased after PI-PLC treatment, albeit significantly (*p* < 0.001) to 7435.0 units. The MFI produced upon MAL-II binding in control and treatment samples differed significantly (*p* < 0.0001) from each other (Fig. 2 and Supplementary Fig. 3). The acrosomal intactness indicator lectin PNA, which binds the asialylated galactosyl (β-1, 3) N-acetylgalactosamine produced MFI of 28,334.0 and 23,075.0 units in the salt-treated and the PI-PLC treated spermatozoa, respectively, whereas the MFI produced by PNA binding in the control spermatozoa was 18,759.0 units. The rise in the MFI values, however, was statistically insignificant for both the treatments (Fig. 2 and Supplementary Fig. 3).

Overall, both the treatments reduced the availability of respective cognate glycans for most lectins except the PNA after salt treatment. Contrarily, the PI-PLC treatment led to increased exposure of α-2, 3 linked sialic acid and asialylated galactosyl (β-1, 3) N-acetylgalactosamine. Furthermore, both the treatments were significantly different from each other *vis-à-vis* the MFI produced upon lectin binding on the surface of the buffalo spermatozoa.

### Expression dynamics of BuBD-129 and 126

**Fig. 3:**
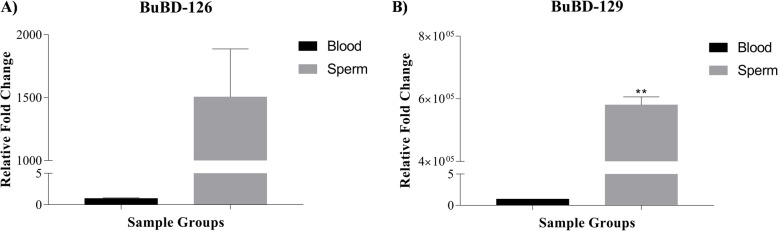
Relative expression profiles of the two CA-BD genes, BuBD-126 **a** and BuBD-129 **b** in the peripheral blood and ejaculated spermatozoa obtained from Murrah buffalo bulls. Expression values were normalized to GAPDH & eEF-2. Horizontal bars represent the mean(s) and error bars represent the standard error of the mean (SEM)

The relative expression profiles of the BuBD129 and 126 genes were generated using RT-qPCR, in the spermatozoa. The expression of the BuBD-126 and BuBD-129 was found to be much higher in the spermatozoa than the peripheral blood (Fig. 3) hinting at their role in buffalo reproduction. Surprisingly this elevated expression of BuBD-126 in blood was found to be non-significant (*p* > 0.05), as assessed by an unpaired two-tailed t-test. Similarly, the expression analysis of BuBD-129 revealed a higher number of transcripts in buffalo spermatozoa relative to the peripheral blood, albeit the mean expression levels between the spermatozoa and the blood were statistically significant (*p* < 0.01). Interestingly a large inter-animal variation was observed in the expression levels of the BuBD129 and 126 amongst the four biological replicates. These observations intriguingly provided a clue that either the spermatozoa actively expressed these BuBDs or their transcripts were already stored in the de-differentiated spermatozoa during spermatogenesis, apart from their preferential expression in the MRT.

### Differential spatial distribution of BuBD-129 and 126

The peptides GRCKEYCNMDEKELDK and NKTGNCRSTCRNGEK for BuBD-129 and BuBD-126, respectively were predicted to be highly antigenic and were thus adjudged as the best B-cell epitopes. This is because they were predicted to be preferentially present in turns and loops and had a comparatively higher probability of being found on the surface (Supplementary Fig. 4). Initially, the crude concentration of the isolated IgGs was assayed by measuring the A_280_ which was 155,049 ng/μl and 168,722 ng/μl for the CA-BDs, BuBD-129 and 126 respectively (Supplementary Fig. 5). Subsequently, Bradford’s assay was used to ascertain the concentration and 0.5μg/ml of the purified antibody was used for further experiments like the IF, IVF studies.

**Fig. 4:**
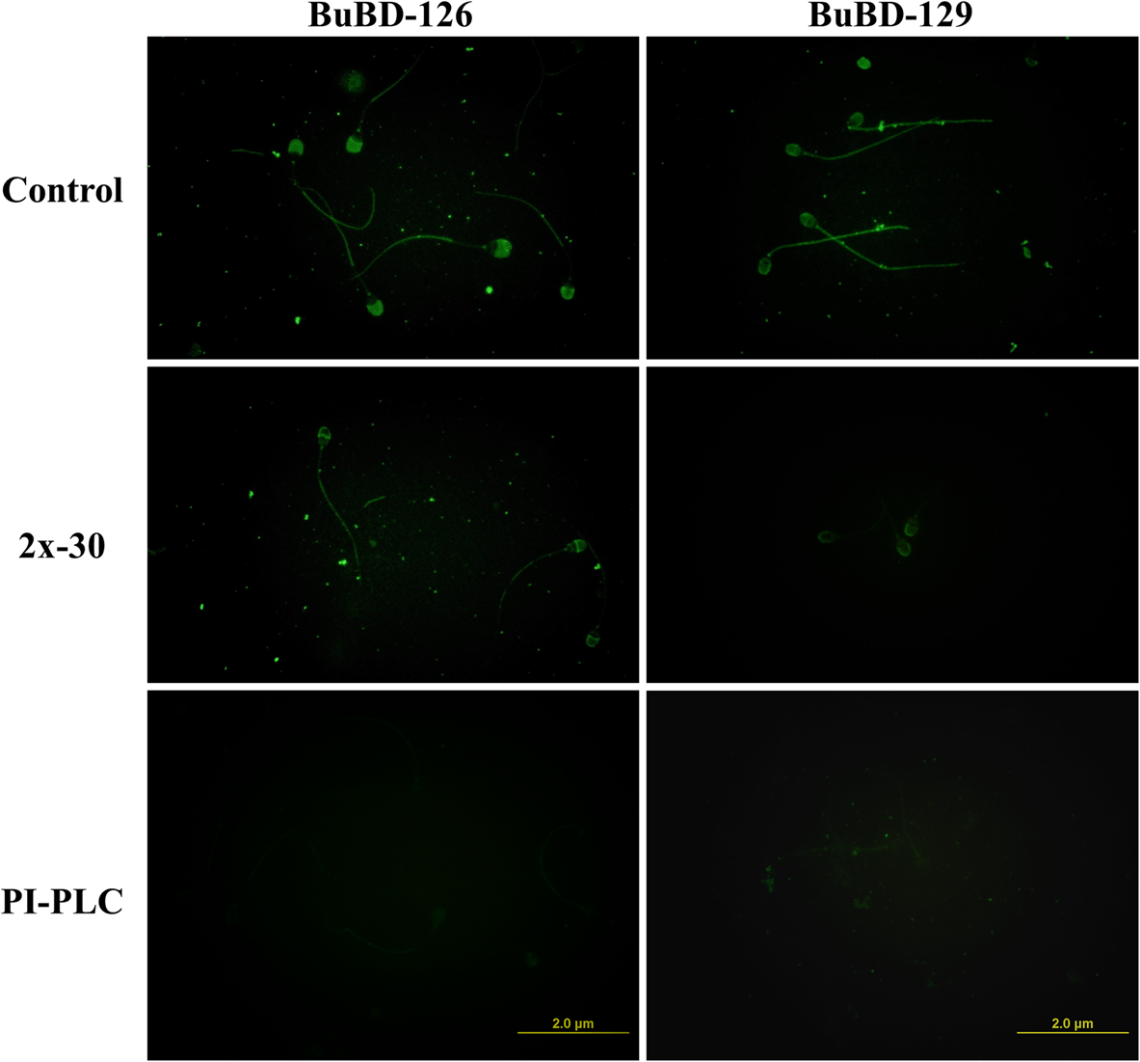
Immuno-localization pattern of the two CA-BDs viz. BuBD-126 and 129 using the in house generated anti-BuBD-129 and 126 antibodies, respectively in rabbit against selected B-epitopes. The decrease of the fluorescent signal intensity pertaining to the removal of the CA-BDs, BuBD-126 and 129 from the buffalo bull sperm surface is observable after both, the 2x-DPBS and PI-PLC treatments

The immunofluorescence (IF) experiments using anti-BuBD-129 and 126 antibodies revealed that the two class-A BuBDs (CA-BDs), BuBD-129 and 126 localized differentially on the surface of the buffalo bull spermatozoa. A variation in the spatial distribution pattern of these two CA-BDs was observed wherein the BuBD-129 was present along the entire periphery of the buffalo spermatozoa (Fig. 4). The BuBD-126, however, was found to be present preferentially on the acrosomal and post-acrosomal and in the tail region while being absent in the mid-piece region (Fig. 4). The negative controls for both the antibodies, which were without the primary antibody did not fluoresce upon excitation (Supplementary Fig. 6). The fluorescence produced by the BuBD-129 and 126 diminished when the spermatozoa were incubated in an elevated salt environment. The spermatozoa incubated in 2x-DPBS for 30 min appear to lose the sperm-surface bound BuBD-126 uniformly from the sperm surface, whereas the BuBD-129 was retained on the acrosomal region) despite being lost from the mid-piece and the tail region of the buffalo bull spermatozoa (Fig. 4). The effect for the PI-PLC treatment, however, was markedly different for the sperm surface-bound BuBD-129. The spermatozoa exposed to 2 U/mL of PI-PLC lost the majority of the fluorescence signal for BuBD-129 from the entire spermatozoa. Nevertheless, the fluorescence pattern for BuBD-126 was similar to what was observed after 2x-DPBS treatment albeit much weaker in intensity indicating higher loss of the bound BuBD-126 (Fig. 4).

### Blocking BuBD-129 on sperm surface hinders cleavage, Morula and blastocyst formation rates

**Fig. 5:**
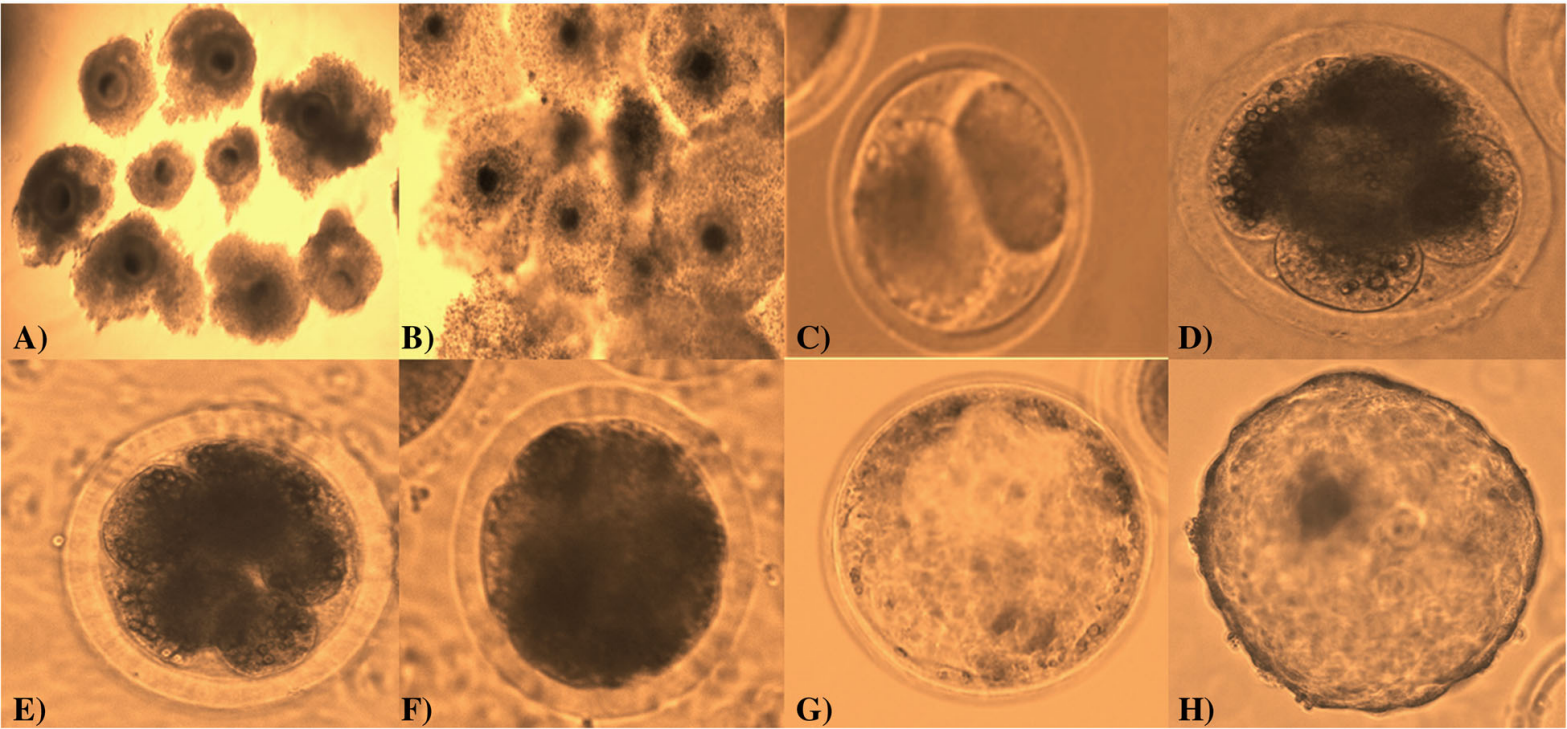
Bright-field images of **a** Grade A and B oocytes aspirated from buffalo ovaries, **b** Matured cumulus-oocyte complexes after IVM, **c** 2-celled stage, **d** 4-celled stage, **e** 8-celled stage, **f** 16-celled stage, **g** Morula and **h** Blastocyst stage of buffalo embryo observed during IVF studies

**Fig. 6:**
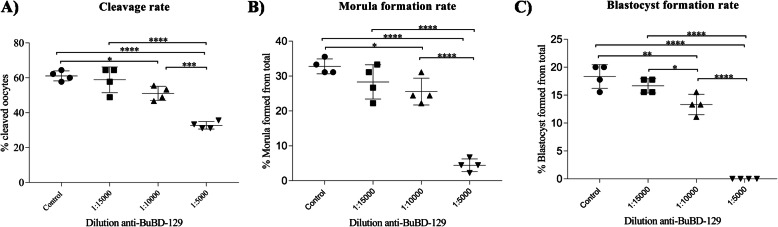
Scatter plots showing the Mean ± SD for cleavage rate **a**, morula **b** and blastocyst formation rates **c** in the control group and samples treated with three different concentrations of anti-BuBD-129

The addition of anti-BuBD-129 antibody in the fertilization medium appeared to hamper the fertilization and thus the subsequent embryonic development in a dose-dependent manner (Fig. 5 and 6). The percentage of cleaved oocytes decreased in the 1:15000 dilution group compared to the control group which further dropped significantly (*p* < 0.05) in the 1:10000 and 1:5000 (*p* < 0.00001) dilution (Fig. 6a). Both the 1:10000 and 1:15000 differed significantly (*p* < 0.001) from the 1:5000 dilution and the control group for the number of cleaved oocytes. The subsequent stages of embryo development e.g. the morula formation also exhibited a similar trend (Fig. 6b). The percentage of morula formed decreased in the 1:15000 dilution but declined significantly (*p* < 0.05) in the 1:10000 dilution which further reduced (*p* < 0.00001) in the 1:5000 dilution group. As expected, the blastocyst formation rate was highest in control which declined (*p* < 0.01) on the addition of anti-BuBD-129 in 1:15000 and 1:10000 (*p* < 0.01) dilution groups (Fig. 6c). No blastocyst was formed in the 1:5000 dilution group.

## Discussion

The present study was designed to identify the proteins associated with the peripheral coats on buffalo spermatozoa which are acquired during their transit through the epididymis and other ducts before ejaculation. The over-represented immune-related glycoproteins and other glycoconjugates of sperm surface peripheral coats are known to regulate male fertility e.g. by assisting in immune-evasion [28, 29]. We sought to specifically remove i) the proteins bound through electrostatic interactions (by elevated NaCl) ii) the GPI-APs (by PI-PLC enzyme). We also wanted to determine if the previously detected epididymal transcripts of buffalo beta-defensins (BDs), the innate immune effectors, are translated and subsequently applied to the buffalo sperm surface. Shotgun proteomic profiling of buffalo sperm-surface proteins revealed that a majority of them are extracellular and are involved in either immune system response or reproductive processes (Table 2). Besides, numerous beta-defensins including the two BDs implicated in male fertility (CA-BDs) i.e. the heavily O-glycosylated BuBD-129 and BuBD-126 were also identified along with other sperm-surface proteins (Supplementary sheet-Results). The in silico prediction of glycosylation of sperm surface proteins was validated, in vitro, by flow cytometry using six lectins (Fig. 2). The presence of BuBD-129 and 126 on buffalo sperm-surface was confirmed by immunofluorescence which revealed a differential spatial distribution pattern of these BDs (Fig. 4). Besides, blocking the BuBD-129 with anti-BuBD-129 antibody was found to hamper the fertilization of buffalo oocytes which subsequently affected embryogenesis (Fig. 6).

The post-gonadal modifications occur chronologically in the epididymal lumen wherein the traversing spermatozoa interact with bio-molecular components in the surrounding milieu.

These biochemical modifications include removal, processing or addition of proteins as well as the changes in the glycans associated with the (glyco) proteins [9, 13, 19, 40, 41]. Broadly, two distinctive and separate populations of proteins have been described on mammalian spermatozoa. The major one of them is adsorbed and loosely adhered onto the sperm-surface and is not integrated into the sperm plasma membrane [3, 19, 42, 43] and. To elucidate such non-covalently bound (ionic) sperm surface antigens, we used elevated salt (NaCl) concentration i.e. 2x-DPBS. Although, it had previously been documented that a population of such non-covalently bound sperm surface (glyco)proteins could be released by exposing the macaque spermatozoa to 2x DPBS i.e. 300 mM NaCl [44] the proteomics profile of these proteins was not available. Many (glyco) proteins, e.g. the cattle PDC-109 and primate DEFB-126, are known to be released from the mammalian sperm-surface after elevated salt or PI-PLC extractions [45–47]. Thus, the other population of sperm-surface proteins is GPI-linked and is known to be firmly integrated into particular microdomains on its plasma membrane. Most of the GPI-APs are laid down on the sperm-surface during epididymal transit of the sperm and these (glyco) proteins have aptly been addressed as ‘maturation antigens [48–51]. We also removed the GPI-APs from the buffalo sperm-surface using the enzyme PI-PLC and subjected them to gene ontology and singular enrichment analysis. Our results revealed that most of the proteins that adorn buffalo sperm-surface (e.g. the BDs) are immune response-related and reproduction-specific (glyco) proteins, as observed in other species [48, 52–54].

Throughout nature, innate immunity and sexual reproduction are tightly linked. As observed for buffalo sperm-surface proteins, a growing body of evidence suggests that a major fraction of sperm-surface proteins belong to the innate defence family and are required for surmounting various immunologic impediments in the FRT [3, 48, 55–60]. Expectedly, the epididymis-specialized genes are thus known to be overrepresented by the genes encoding secretory proteins which are involved in the innate immune response and reproduction [16, 56, 61–63]. Many molecules e.g. the highly glycosylated and negatively charged, antimicrobial peptides, beta-defensins (BDs) have been discovered in the lumen of the mammalian epididymis that help the spermatozoa in their survival and performance the male reproductive tract (MRT) and FRT [64–66]. The identification of many BDs including the two CA-BDs viz. BuBD-129 and 126 indicated that the translated products of these genes are applied as peripheral coats onto the buffalo sperm surface, as reported in primates and other ruminants [29, 51]. Recently, many studies that link the BDs with the regulation of male fertility have been reported in multiple mammalian species [30, 38, 45, 51, 67–69]. Interestingly, the number of BD genes and thus the epididymal secreted BDs found on sperm, are highly variable in different species at least partly, due to the differences in microbial load, historical contingency, genetic drift and disparate ecological niches occupied [64, 70, 71]. The epididymal BDs found on buffalo sperm surface are among the class of proteins that are weakly bound, presumably by non-covalent (ionic) interactions. As observed in buffalo, many BDs e.g. DEFB-126 and DEFB-15 are known to be expressed and secreted by the epididymal epithelium cells which subsequently interact with the traversing spermatozoa surface across mammalian species [43, 51, 66, 72, 73]. The BDs are amongst the dominant molecules of the buffalo sperm surface apparently due to their heavy glycosylation, their ability to interact with phospholipid membranes and their immunologic activity [9, 13, 29, 30, 39].

The glycocalyx of the sperm is the molecular frontier that interacts directly with the hostile and immunologic milieu of the FRT. The flow cytometry experiments not only validated the presence of the cognate glycans for the six lectins on the sperm-surface but also helped to monitor the reduction in the O-linked and N-linked glycans from buffalo sperm-surface after protein extraction treatments. We have previously demonstrated that the BuBD-129 is an atypical BD, possessing a long C′ tail and was predicted to be heavily O-glycosylated, similar to what has been reported for the primate DEFB-126 [29, 32, 74]. Other epididymal secreted BDs like murine Defb-15 have also been predicted to be heavily O-glycosylated and has a long 20 amino-acid extension in the C′ of its protein [42]. Our results thus suggested that the glycocalyx barrier on the buffalo spermatozoa is either removed or greatly re-organized post the elevated salt or PI-PLC treatments. Therefore, the cognate glycans for ABL, LEL and JAC appeared to be presented in a way to become unavailable for lectin binding after these treatments. Interestingly, the recognition of negatively charged, terminally positioned, sialic acids by MAL-II on the buffalo sperm surface pointed towards their role as a protective coat. This is because the sialic acid moieties are known to bestow upon the spermatozoa the ability to evade the elicited immune responses in the FRT [29, 32, 46]. Alternatively, they mask the testicular protein components making the sperm invisible to the FRT’s immune-surveillance [3, 29, 75]. Although the reduction in the MFI signal indicated either the deletion or conformational change in the available glycans this approach can’t pinpoint individual sperm glycoprotein rather identifies general shifts in the surface sugars on the sperm-surface.

Immunofluorescence (IF) revealed a differential spatial distribution pattern of the heavily O-glycosylated peripheral protein BuBD-129 and BuBD-126 on the buffalo sperm surface. The BuBD-129 spanned the entire buffalo spermatozoa similar to what has been observed for the primate DEFB-126 and rodent defb22 [44, 76, 77]. Although the coats of maturation antigens and their interacting partners tend to localize to specific micro-domains, nevertheless, few of them such as BuBD-129, rodent defb22 and the primate DEFB-126 are adsorbed globally around the entire spermatozoa [29, 40, 78, 79]. This similarity in the spatial distribution pattern hints at the existence of functional orthology between the buffalo BuBD-129, rodent defb22 and primate DEFB-126, apart from other similarities like gene length, chromosome cluster, an extended amino-acid tail at C-terminus, a high potential of O- and N-glycosylation and the preferential expression in the distal segments of MRT [74]. The peripheral localization of BuBD-129 along the entire buffalo spermatozoa perimeter presumably establishes a barrier between the buffalo spermatozoa and the immunologic milieu of the FRT as reported for other BDs in other mammalian species [3, 29, 50, 75]. Despite being identified amongst the dominant proteins on the buffalo sperm surface, the BuBDs were not deemed as antigenic sperm-surface proteins by the female immune system. This indicates their role in immune-protection because the antibodies were generated against other proteins (e.g. Acrosin and Profilin) rather than the BuBDs when female Wistar rats were immunized with buffalo sperm-surface extracted proteins (V Batra, R Kumar and TK Datta, Unpublished data). On the other hand, the spatial distribution of BuBD-126 and BBD-126 [67] differs from the observed binding pattern of DEFB-126 in monkeys [78] or defb22 in rodents [79]. The BuBD-126 was found to localize to the post-acrosomal region and the tail rather than environing the whole sperm surface like the primate DEFB-126 and defb22 in mice. These species-specific differences could be ascribed to the high variability in the distribution pattern of glycoconjugates and thus in the manifested Sperm Associated Glycan Topography (SpAGT) in various species [28].

IVF was used to predict the reproductive functional significance of the BuBDs. The blocking of BuBD-129 reduced the cleavage as well as the morula and blastocyst formation rate of the fertilized buffalo oocytes during IVF. It is well established that the antibodies directed against the sperm-specific antigens (ASAs) are a major cause of immunological infertility since they perturb the normal fertilization process [3, 50, 80]. Blocking the BuBD-129 appeared to preclude successful fertilization events which confirmed their presence on buffalo sperm-surface. Likewise, the ortholog of primate DEFB-126 in mice (defb22) has been demonstrated to incorporate in the oolemma during gamete fusion which subsequently floated and extended out from the fused spermatozoa [81, 82]. Besides, its ortholog in cattle (BBD-126) has been reported to be retained on the sperm surface, after induction of in vitro capacitation [67]. This suggests that the BBD-126 too remains associated with the spermatozoa during fertilization indicating an additional role in fertilization [47]. It has been proposed that these features may be true for all the BDs with high levels of O-glycosylation [83]. Our results indicated that the BuBD-129 is present on the buffalo sperm-surface even after induction of in vitro capacitation. Similar to BuBD-129, antibodies against another member of the CAP superfamily, CRISP1, a sperm-surface protein have been demonstrated to obstruct fertilization by interfering in the sperm-oocytes fusion process, thus reducing fertility [78, 84]. The cysteine-rich defensin-like peptides appear to be integral to the reproductive success of organism ranging from invertebrates, plants to higher primates [85–88]. It cannot be denied that the blocking of BuBD-129 changes the attributes of sperm surface features which not only influence fertility but also the developmental potential of the subsequent embryos. The BuBD-129 probably imparts surface properties that are essential for fertilization by the buffalo sperm due to its heavy O-linked glycosylation and uniform spatial distribution on the entire buffalo sperm. These results suggest multiple and putatively epistatic roles of the BD genes in immune response and reproductive physiology of buffalo, e.g.in the process of sperm-oocytes interaction.

## Conclusion

The buffalo sperm surface is heavily glycosylated and many glycoproteins are applied as peripheral coats on to the surface of mammalian spermatozoa. Many of these glycoproteins are immune-regulatory and have reproduction-specific functions. The molecular functions and biological roles of only a limited number of such proteins have been studied regarding their role in male reproductive physiology. The BuBDs like BuBD-129 and 126 are amongst the dominant molecules of the buffalo sperm surface. It would be interesting to quantify the BuBD-129 abundance in LF and HF bulls with contrasting field CRs through a targeted proteomics approach. The effect of exogenous supplementation of recombinant BuBD-129 to the low fertile sperm to augment the current field fertility rates should also be evaluated. Further investigations into the functional roles of this critical component of buffalo sperm are warranted.

## Methods

### Chemicals and plasticware

Chemicals and media used in the present study were obtained from Sigma Chemicals Co., St. Louis, Missouri, USA/ Qiagen/Fermentas/Invitrogen as mentioned for specific cases.

### Semen collection and pre-processing

Freshly ejaculated normozoospermic semen samples (mass motility ≥3, *n* = 5–12, aged between 3 and 5 years) were collected from mature Murrah buffalo bulls (of proven fertility) using the artificial vagina at the Artificial Breeding Research Centre (NDRI, Karnal, India). Each ejaculate was collected in a 15 mL centrifuge tube containing twice the volume of non-capacitating media (NCM), Sp-TALP i.e. HEPES buffered Tyrode’s medium (pH 7.4, 37 °C). The semen was transported to the laboratory at 37 °C and was washed thrice with Sp-TALP by centrifuging at 280 x g for 6 min to remove the seminal plasma and its protein components. The motile spermatozoa were selected by subjecting the final pellet to the swim-up technique. The upper 1.5 mL volume was later collected and centrifuged to obtain the pellet of motile spermatozoa.

### Surface protein extraction from motile spermatozoa

#### Elevated salt extraction of surface proteins

The post-swim-up spermatozoa in Sp-TALP were washed in DPBS and divided into four groups by re-suspending in either 2x or 4x DPBS for 30 min and 60 min at 37 °C (viz. 2x-30, 2x-60, 4x-30 and 4x-60). The micro-centrifuge tubes were shaken gently during the period of incubation and the samples were subsequently pelletized by centrifugation at 280 x g for 10 min. A pool of supernatants from 5 to 7 ejaculates was collected and subsequently filtered through a 0.22 μm filter. The proteins in the filtrate were precipitated with acetone precipitation method (1:9, supernatant/acetone ratio), concentrated on a speed-vac vacuum concentrator and subjected to SDS-PAGE after quantification by Quick Start™ Bradford protein assay.

#### PI-PLC extraction of GPI-APs

The PI-PLC enzyme specifically hydrolyzes the phosphodiester bond of phosphatidylinositol to form a free 1, 2-diacylglycerol and glycopeptide-bound Myo-inositol 1,2-cyclic phosphate and is therefore used to release the GPI-APs from the surface of the membranes [44, 77]. The post-swim-up spermatozoa (100 × 10^6^) in Sp-TALP were incubated with three different concentrations of Phosphoinositide-phospholipase C (PI-PLC) from *Bacillus cereus* (1 U/mL, 1.5 U/mL and 2 U/mL) in siliconized tubes at 37 °C for 2 h. The siliconized tubes were shaken gently during the period of incubation. Thereafter, the samples were centrifuged at 1000 x g for 10 min and the supernatants were collected and then filtered through a 0.22 μm filter. The supernatants from 10 to 12 ejaculates were pooled and then precipitated by acetone precipitation method and quantified before subjecting to SDS-PAGE.

### Mass spectrometry (LC-MS/MS) of sperm-surface extracted proteins

**Fig. 7:**
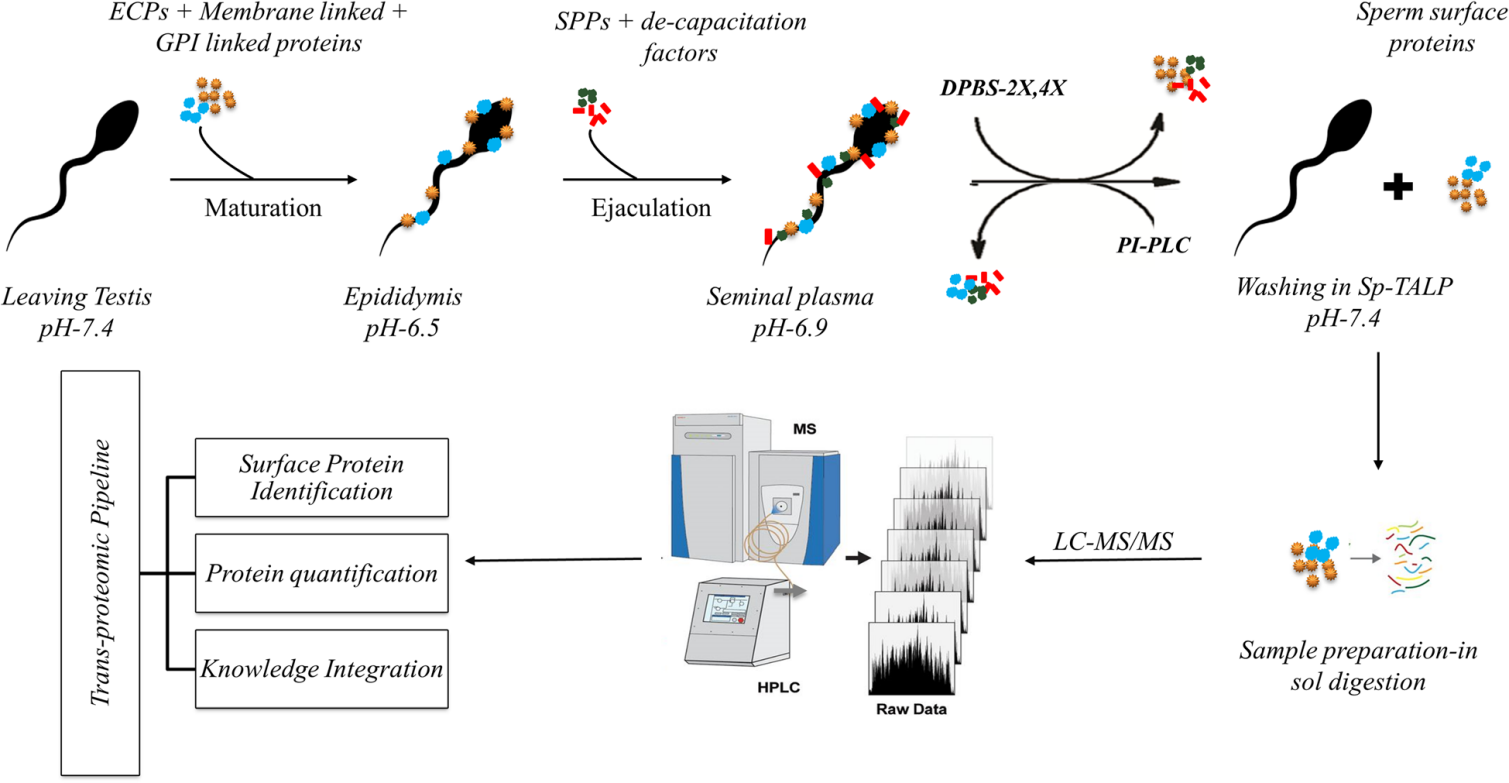
The overall research methodology followed for BuBD identification on the buffalo sperm surface by LC-MS/MS.

For the identification of sperm surface proteins, mass-spectrometry was performed using the method described by Gourinath et al. [89]. The extracted proteins (100 μg) from the pooled elevated salt and PI-PLC extractions were dissolved in 6 M guanidium hydrochloride. Subsequently, 25 μL of the dissolved samples were reduced with 5 mM tris (2-carboxyethyl) phosphine (TCEP). The samples were then alkylated with 50 mM iodoacetamide for 20 min in dark at room temperature (RT) and then digested with trypsin (1:50, trypsin/lysate ratio) for 16 h at 37 °C after re-suspension in digestion buffer. The digests were cleaned using a C18 silica cartridge to remove the salt and dried using a speed vac vacuum concentrator. The dried pellet was resuspended in buffer A (5% acetonitrile, 0.1% formic acid). All the experiments were performed using EASY-nLC 1000 system (Thermo Fisher Scientific), which was coupled to a QExactive Mass Spectrometer (Thermo Fisher Scientific) equipped with a nano-electrospray ion source. One μg of the peptide mixture was resolved using a 25 cm PicoFrit column (360 μm outer diameter, 75 μm inner diameter, 10 μm tip) filled with 1.8 μm of C18-resin (Dr Maisch, Ammerbuch, Germany). The peptides were loaded with buffer A and eluted with a 0–40% gradient of buffer B (95% acetonitrile, 0.1% formic acid) at a flow rate of 300 nL/min for 90 min. The MS data were acquired using a data-dependent top 10 method dynamically choosing the most abundant precursor ions from the survey scan (Fig. 7). All raw MS data have been deposited to the ProteomeXchange consortium through the PRIDE partner repository (Identifier: PXD022114).

### LC-MS/MS data processing

The generated raw files for the seven samples were analyzed using Trans-Proteomic Pipeline TPP v5.1 (Syzygy) rev.0 [90], against a database generated from UniProt knowledgebase (*Bos taurus*, *Homo sapiens*, *Bubalus bubalis* and beta-defensin, downloaded January 3, 2019; www.uniprot.org) using the Comet search engine [91]. The precursor and fragment mass tolerances were set at 10 ppm and 0.5 Da, respectively. The enzyme specificity was set to trypsin/P and a maximum of two missed cleavages were allowed. Carbamidomethyl on cysteine (C) was considered as fixed modification while oxidation of methionine and N-terminal acetylation were considered as variable modifications for the database search. The peptide spectrum match and protein false discovery rates (FDR) were set to 0.01 to increase the confidence and remove the false-positive identifications. The use of iProphet tool in TPP results in an increased number of correctly identified peptides at a constant FDR because it combines the evidence from multiple identifications of the same peptide sequences across different spectra, experiments, precursor ion charge states, and modified states. Only the proteins with iProphet probability greater than 0.99 were considered for further analysis [92].

### Singular enrichment and scatter plot analyses of the identified proteins

The gene-ontology and singular enrichment analysis for identified proteins in all of the seven treatments were performed using agriGO [93] which is a specialized GO analysis toolkit and database for livestock species relevant to the agricultural community. For SEA (Singular Enrichment Analysis), the Bovine genome locus (Bovine Genome Database): GLEAN_03528 was used as background dataset and Fisher’s test was used to calculate the *p*-values. The Yekutieli (FDR under dependency) method [94] was used for Multi-test adjustment. The minimum number of mapping entries for analysis was set to 5 and values with *p*⩽0.05 were considered significant. The resulting long lists of Gene Ontology terms were summarized by REViGO webserver [95] which removes the redundant terms and the remaining terms were visualized in semantic similarity-based scatterplots. The SimRel was used as the semantic similarity measure (clustering algorithm) against the whole Uniprot database which employs a multidimensional scaling procedure that initially places the terms using eigenvalue decomposition of the terms’ pairwise distance matrix. This was followed by a stress minimization step which iteratively improves the agreement between the GO terms’ semantic similarities and their closeness in the displayed two-dimensional space.

### Flow cytometry

The validation of the surface (glyco) protein removal after elevated salt and PI-PLC treatments was done by flow cytometry using six lectins viz. ABL, LEL, JAC, MAL-II, LCA and PNA (Table 3), which bind to various cognate glycans on the sperm surface, including an unstained sample as the negative control (*n* = 3). A protocol for sperm flow cytometry analysis standardized by Batra et al. [28] was followed for signal acquisition. Briefly, the sperm were washed as described earlier and the concentration was adjusted to 3 × 10^6^ sperm/ml. Samples were incubated with lectins for 10 min at 38.6 °C under an atmosphere of 5% CO_2_ before the flow cytometry analysis which was performed with a standard bench-top BD Accuri C6 flow cytometer (Becton Dickinson Biosciences, Ann Arbor, MI, USA, with BD Accuri C6 software v.1.0.27.1). The cytometer was calibrated daily according to the manufacturer’s recommendations with 8 and 6 peak calibration beads and QC was performed every second day using BD CS&T RUO beads. The 488-nm laser was used for the excitation of FITC, and its emission was filtered using a 533/30 bandpass filter. Filtered emissions were detected by photomultiplier tubes. A threshold of 80,000 in the forward scatter (FSC) signal was applied to remove electrical noise, and very small events and samples were acquired at a low flow rate (14 μl/min). For each sample, 20,000 events (single cells) were acquired. Only the singlet population was identified and used for analyses. The statistical analyses were performed on Prism Graphpad 7.0 (for Windows, GraphPad Software, La Jolla California USA, www.graphpad.com) by one way ANOVA to test the difference of means and the Brown-Forsythe test to test the differences in the standard deviations of the MFI values produced from various FITC-bound lectins.

**Table 3:** The six lectins used to assess changes in the sperm-surface after salt/PI-PLC treatments

Lectin	Major sugar recognized
LEL (Lycopericon esculentum lectin)	[GlcNAc]1–3, N-acetylglucosamine
ABL (Agaricus bisporus lectin)	Galactosyl (β-1,3) N-acetylgalactosamine
JAC (Jacalin)	Mono or di-sialylated ofT-antigen
MAL II (*Maackia amurensis* lectin II)	α-2,3 linked Sialic acid
LCA (*Lens culinaris* agglutinin)	Mannose and Glucose moieties
PNA (Peanut agglutinin)	Asialylatedgalactosyl (β-1,3)N-acetylgalactosamine

### Expression dynamics of the BuBD-126 and BuBD-129 in the buffalo spermatozoa

Total RNA was isolated from the peripheral blood and the spermatozoa as described previously [96, 97] using the TRI Reagent, RNA isolation reagent (Sigma-Aldrich, USA) and was quantified using a NanoDrop ND-1000 UV–Vis spectrophotometer (NanoDrop Technologies Inc., Wilmington, DE, USA). The A260/280 and A260/230 were close to 2.0 for all the samples used in the study. The quality of the extracted RNA was assessed by running 200 ng of the RNA (heated at 70 °C for 1 min) in non-denaturing TAE buffered 1.2% agarose (Sigma-Aldrich, USA). The cDNA synthesis and RT-qPCR optimization were done as described by Batra et al. [74]. The MIQE [98] guidelines were followed at every step, wherever possible. The relative quantification of the BD genes was done on a Bio-rad CFX-96 Touch Deep Well Real-Time PCR system platform using the iTaq Universal SYBR Green Supermix (Bio-Rad, USA) in a10 μL reaction mix. The thermal profile was 95 °C for 5 min, 40 cycles consisting of denaturation at 95 °C for 15 s, annealing at variable optimized temperatures for 20sand extension at 72 °C for 20 s, followed by the melt curve protocol with 10 s at 95 °C and then 60 s each at 0.5 °C increments between 65 °C and 95 °C. A no-template control (NTC) was run in each plate to confirm the absence of nucleic acid contamination. A melt curve analysis was performed to ensure a specific, unique product formation and to ascertain minimal primer dimer formation. The mean sample Cq (Cycle of quantification) values for the BuBD-126 and 129 were calculated for duplicate samples and their relative expression was calculated as described previously [41]. The GAPDH (glyceraldehyde-3-phosphate dehydrogenase) and eEF-2 (eukaryotic elongation factor) were used as the reference genes, and the blood was considered as the calibrator for the quantification of gene expression. The differential gene expression levels between the blood and the spermatozoa were examined for normality of distribution and were analyzed by an unpaired two-tailed t-test as implemented in GraphPad Prism 7.0 (for Windows, GraphPad Software, La Jolla California USA, www.graphpad.com) and a *p*-value < 0.05 was considered to be statistically significant.

### Antibody development for determining the spatial distribution pattern of BuBD-129 and 126

A custom polyclonal antibody specific for BuBD-129 and 126 was commercially generated by Genei using a standard protocol. Briefly, the amino acid peptide sequences from the secreted fragments of BuBD-129 and 126 were selected based on computational modelling on IEDB [99] for being surface epitopes. After chemical synthesis and conjugation to keyhole limpet hemocyanin (KLH), the epitope (500 μg mixed with Freund’s complete adjuvant) was inoculated subcutaneously into the back of the neck region of sexually mature nulliparous female New Zealand white rabbits (*n* = 1, each group) weighing between 1.1–1.5Kg (1 animal per cage). The pre-immune sera (control) were extracted from the blood taken from the central ear vein by the skilled technicians in the presence of a veterinary doctor. For initial immunization, 500 μg of either BuBD-129 or 126 KLH-conjugated peptide was mixed in 500 μl of Freund’s complete adjuvant and was administered subcutaneously in the back of the neck region of the animal. However, for booster doses 300 μg of the antigen mixed in 500 μl of Freund’s incomplete adjuvant and was administered similarly. An immune response was confirmed by binding of the serum to the antigen on an enzyme-linked immunosorbent-type assay. The antibody titer was checked using western blot and seven booster doses were deemed necessary to obtain working IgGs isolated from the serum. The blood was collected by bleeding the rabbit through the central ear vein by trained individuals in the presence of a veterinary doctor. The whole blood (5 ml) was subsequently collected from the central ear vein and allowed to clot at RT for 30 min. The serum was isolated from the blood and Protein G based NAb™ Spin Kits were used for isolation of IgG antibodies from the collected serum according to the manufacturer’s instructions. The subsequent quantification of BuBD-129 and 126 IgG antibodies from the serum was performed by measuring the A_280_ of each eluate fraction on an Infinite® 200 NanoQuant microplate reader (Tecan). A confirmatory SDS-PAGE was performed to validate the isolated IgGs.

### Immunofluorescence

The swim-up fraction of spermatozoa (40 x 10^6^cells/ml) was re-suspended in NCM and was added onto poly-L-lysine coated slides. The NCM was removed after 15 min since the spermatozoa adhered to the slide surface by that time. Subsequently, the cells were washed twice in PBS and fixed in 2% paraformaldehyde and 0.1% glutaraldehyde for 20 min at RT. The movement of the GPI-APs is prevented by using low glutaraldehyde concentration. The spermatozoa were then washed with PBS thrice, and the slide surface was then blocked with blocking buffer (1% BSA in PBST-0.1% Tween 20 in 1X-PBS) for 1 h at room temperature. The cells were later incubated with the primary polyclonal antibody (1:1000 dilutions) against BuBD-129 and 126 overnight at 4 °C. The sperm were washed with PBST thrice and were then incubated with FITC conjugated goat anti-rabbit IgG secondary antibody (1:5000 dilutions; Sigma-Aldrich) in dark for 1 h at RT followed by final washings with PBST (3x). After the final washing, a coverslip was mounted onto a glass slide onto which one drop of mounting medium, Dabco® 33-LV was placed. The cells were then observed under a BX-51 Olympus fluorescence microscope. The loss of the BuBD-129 and 126 from the sperm-surface after incubation of spermatozoa in 2x-DPBS or exposing them to 2 U/ml of PI-PLC was similarly monitored by immunofluorescence after the stipulated time.

### IVF study

Buffalo ovaries were collected from a local abattoir, Delhi regardless of the oestrous cycle stage and were transported within 2-3 h to the laboratory in physiological saline (0.9%, w/v NaCl) containing strepto-penicillin (50 mg/l). Ovaries were washed 3–4 times in normal saline and the cumulus-oocyte complexes (COCs) were aspirated from the follicles with the help of a vacuum aspiration unit (K-MAR-5200 IN, USA) in Hepes-buffered hamster embryo culture (HH) medium and the aspirated follicular fluid was placed in a dry bath at 37.5 °C for 35–40 min. The quality of COCs was observed and graded into grade A (> 5 layer cumulus layer) and B (3–5 cumulus layers) under the stereo-zoom microscope. The COCs of only grade A and B were selected for in vitro maturation (IVM) and in vitro culture (IVC). To perform IVM, the selected COCs were washed 3–4 times with maturation media (HEPES buffered TCM199 modified with 10% (v/v) fetal bovine serum (FBS), 0.005% (w/v) streptomycin, 0.01% (w/v) sodium pyruvate and 0.005% (w/v) glutamine supplemented with 5.0 μg/ml FSH and 10 μg/ml LH, 1 μg/ml estradiol 17-β and 50 ng/ml epidermal growth factor (EGF), 64 μg/ml cysteamine and 50 μl ITS). After washing the COCs were placed in a group of 15 in 100 μl of maturation medium drops. Three such drops were placed in a 35 mm culture dish which was then overlaid with mineral oil dressing. The dishes were cultured in duplicate for 24 h at 38.5 °C in a humidified atmosphere of 5% CO_2_ in an incubator. The IVF was carried out in 100 μl droplets of BO medium supplemented with 1% (w/v) bovine serum albumin (BSA), 1.9 mg/ml caffeine sodium benzoate, 0.14 mg/ml sodium pyruvate and 0.01 mg/ml heparin. For the IVF, the control group didn’t contain anti-BuBD-129 antibody whereas, the three treatment groups comprised of three different concentrations of anti-BuBD-129 (0.5 mg/ml) antibody in the fertilization medium drops viz. the 1:15000, 1:10000 and 1:5000 dilution groups were prepared. The matured COCs were washed thrice in BO medium and placed in BO medium droplets supplemented with the anti-BuBD for treatment groups. The frozen buffalo semen was thawed simultaneously and subsequently processed for in vitro capacitation as per the procedure described earlier by Jain et al. [97]. Fifty μl of BO media was removed from the respective IVF medium drops and 50 μl of the sperm suspension (1 × 10^6^ spermatozoa/mL) was added to each fertilization drop with 15 COCs and was then incubated at 38.5 °C with 5% CO2 for 12 h. The presumptive zygotes were removed from the fertilization drops after 12 h of insemination and the adhered cumulus cells were mechanically removed by vortexing and were washed five times in a modified Charles and Rosenkrans 2 amino acid (mCR2aa) medium. Following washing, the 15 presumptive zygotes were co-cultured with the monolayer of granulosa cells in 100 μl drops of IVC-I medium (mCR2aa supplemented with 0.8% (w/v) BSA, 1 mM glucose, 0.33 mM pyruvate, 1 mM glutamine, 1 x MEM essential amino acid, 1x non-essential amino acid and 50 μg/ml gentamycin). After 48 h post insemination (hpi) zygotes were evaluated for evidence of cleavage. At 72 hpi, all the cleaved embryos were transferred to IVC-II medium (same as IVC-I with 10% FBS) and were maintained for 8 days post insemination at 38.5 °C with 5% CO_2_. The culture medium was replaced regularly at an interval of 48 h. A total of four biological replicates were used for the IVF experiments. The data were analyzed on GraphPad prism7 (for Windows, GraphPad Software, La Jolla California USA, www.graphpad.com) to compare the observed differences in cleavage and blastocyst rates among the control and treatment groups.

## Supplementary Information

**Additional file 1: Supplementary Figure 1.** The extracted sperm-surface proteins. PAGE profiles of the sperm-surface proteins extracted by PI-PLC and elevated salt treatment

**Additional file 2: Supplementary Figure 2.** The correspondence between the extraction treatments. The common sperm-surface proteins identified within salt extraction (A) and PI-PLC treatments (B) and between the two treatment classes (C).

**Additional file 3: Supplementary Figure 3.** The removal of glycans after salt and PI-PLC treatment. Overlay of the MFI histograms obtained by flow cytometry analysis of control, elevated salt and PI-PLC treated spermatozoa from buffalo bulls (*n* = 3 incubated with six FITC-labelled lectins viz. ABL (A) JAC (B) LEL (C), LCA(D), MAL-II(E) AND PNA(F).

**Additional file 4: Supplementary Figure 4.** Surface epitopes make better antigens. B-epitope mapping for BuBD-126 (A) and 129 (B) illustrating the results from IEDB server’s collection of tools and methods

**Additional file 5: Supplementary Figure 5.** The purified IgGs from rabbit serum. Reducing SDS PAGE of the affinity-purified IgGs depicting the major bands near 25 kDa and 50 kDa, corresponding to the light chain (L) and heavy chain (H) of the IgG antibody.

**Additional file 6: Supplementary Figure 6.** Negative controls for BuBD-129 and 126. Bright field and fluorescent micrographs of the negative controls for the anti-BuBD-126 and 129 primary antibodies.

**Additional file 7: Supplementary sheet-Results.** Identified sperm-surface proteins and their gene ontology, scatter plot analysis. The sperm-surface proteins extracted by seven treatments, their Physico-chemical properties and tabulated results of gene ontology and scatter plot analysis.

## Data Availability

All data generated or analysed during this study are included in this published article and its supplementary information files. The LC-MS/MS data are available via ProteomeXchange with identifier **PXD022114.** The flow cytometry datasets generated from this study can be found in the FlowRepository **(Rep ID: FR-FCM-Z3BX).**

## Abbreviations

ABL: *Agaricus bisporus* lectin
AMPs: Antimicrobial peptides
BD/ DEFB: Beta-defensin
BuBD: Buffalo beta-defensin
CA-BD: Class-A beta-defensin
CMP: Cervical mucus penetration
CR: Conception rate
GPI: Glycosylphosphatidylinositol
FRT: Female reproductive tract
IF: Immunofluorescence
JAC: Jacalin
LCA: *Lens culinaris* agglutinin
LC-MS/MS: Liquid chromatography, tandem mass spectrometry
LEL: *Lycopericon esculentum* lectin
MAL II: *Maackia amurensis* lectin
MRT: Male reproductive tract
OEC: Oviductal epithelial cells
PI-PLC: Phosphoinositide-phospholipase C
PNA: Peanut agglutinin
SEA: Singular enrichment analysis
SPA: Scatter Plot analysis
SSR: Sperm surface remodelling
