# Supplementary figures and images for: Buffalo sperm surface proteome profiling reveals an intricate relationship between innate immunity and reproduction
Source: BMC Genomics. 2021 Jun 26;22:480. doi: 10.1186/s12864-021-07640-z (PMC8235841; doi:10.1186/s12864-021-07640-z)

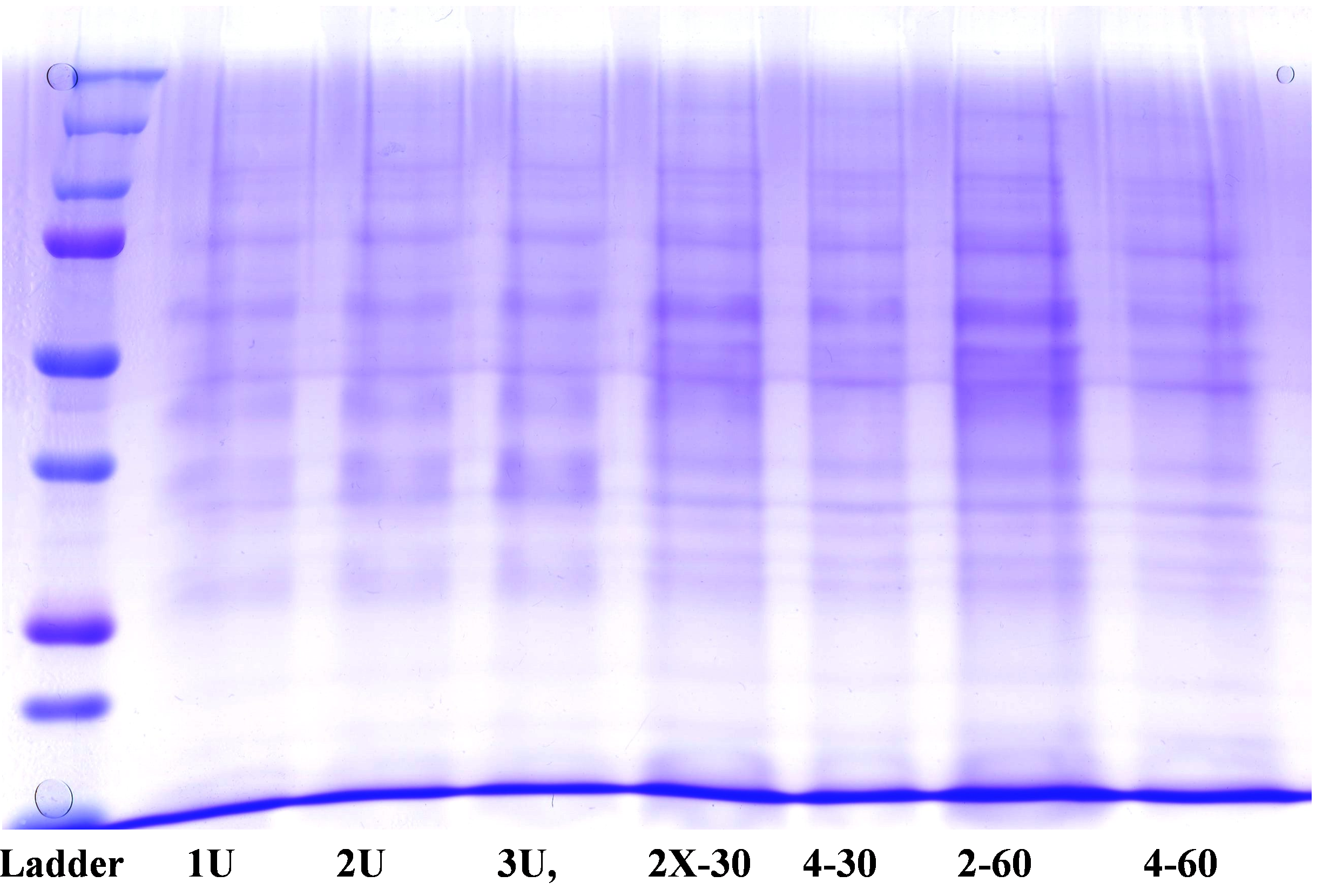

Supplement: Supplementary file 1 — Additional file 1: Supplementary Figure 1. The extracted sperm-surface proteins. PAGE profiles of the sperm-surface proteins extracted by PI-PLC and elevated salt treatment [file 12864_2021_7640_MOESM1_ESM.tif]

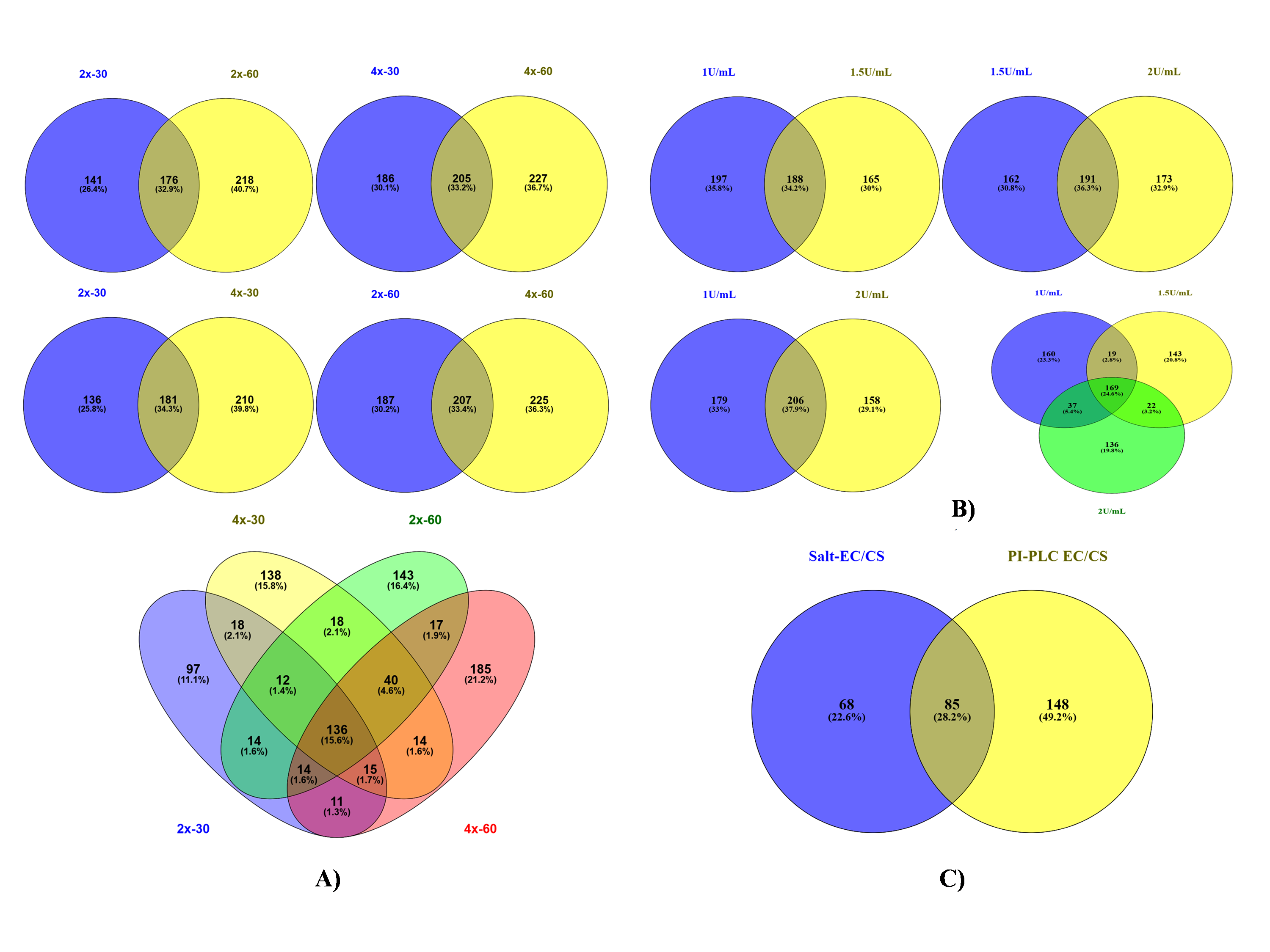

Supplement: Supplementary file 2 — Additional file 2: Supplementary Figure 2. The correspondence between the extraction treatments. The common sperm-surface proteins identified within salt extraction (A) and PI-PLC treatments (B) and between the two treatment classes (C). [file 12864_2021_7640_MOESM2_ESM.tif]

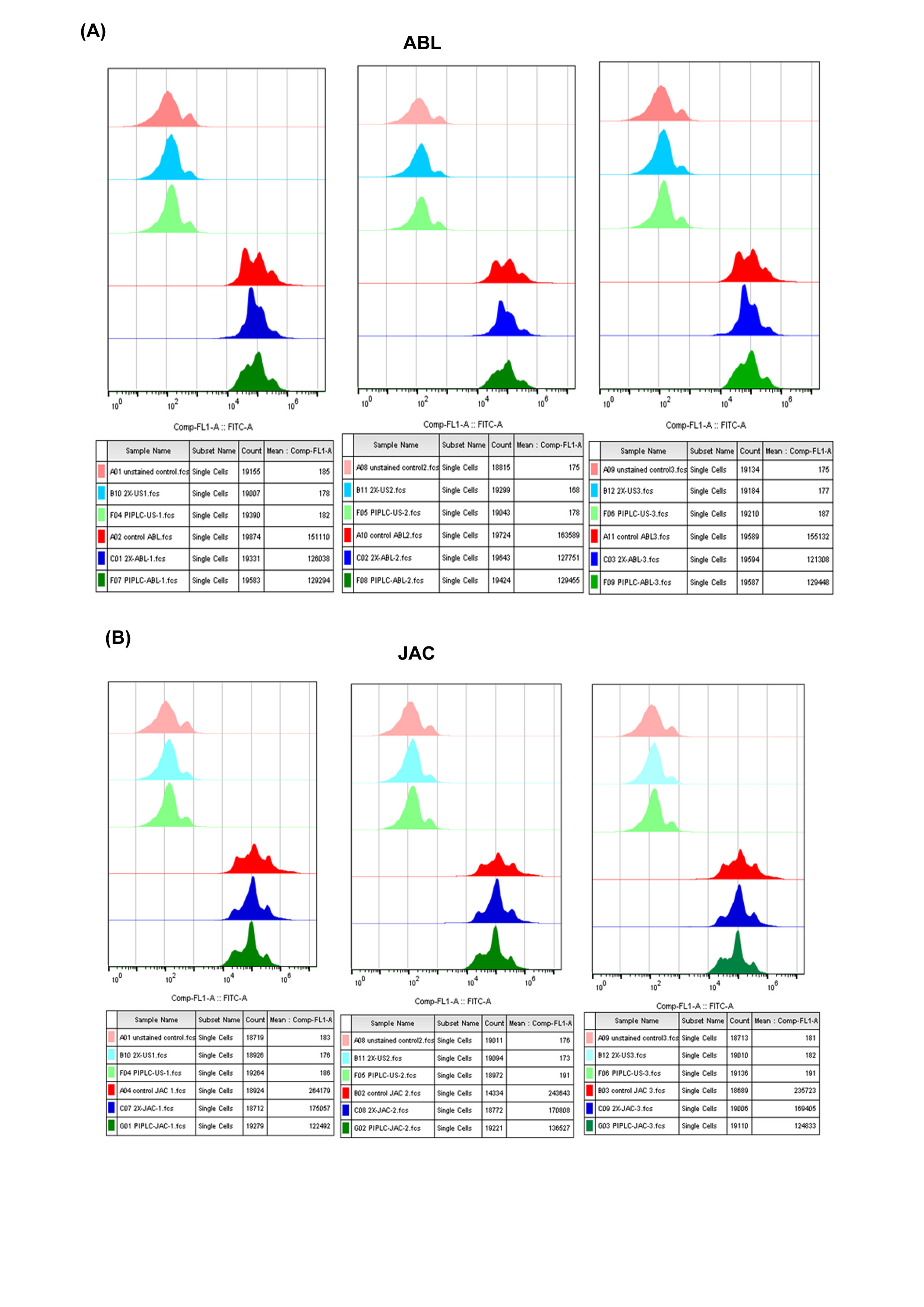

Supplement: Supplementary file 3 — Additional file 3: Supplementary Figure 3. The removal of glycans after salt and PI-PLC treatment. Overlay of the MFI histograms obtained by flow cytometry analysis of control, elevated salt and PI-PLC treated spermatozoa from buffalo bulls (n = 3 incubated with six FITC-labelled lectins viz. ABL (A) JAC (B) LEL (C), LCA(D), MAL-II(E) AND PNA(F). [file 12864_2021_7640_MOESM3_ESM.zip › Supplementary Fig. 3-1.jpg]

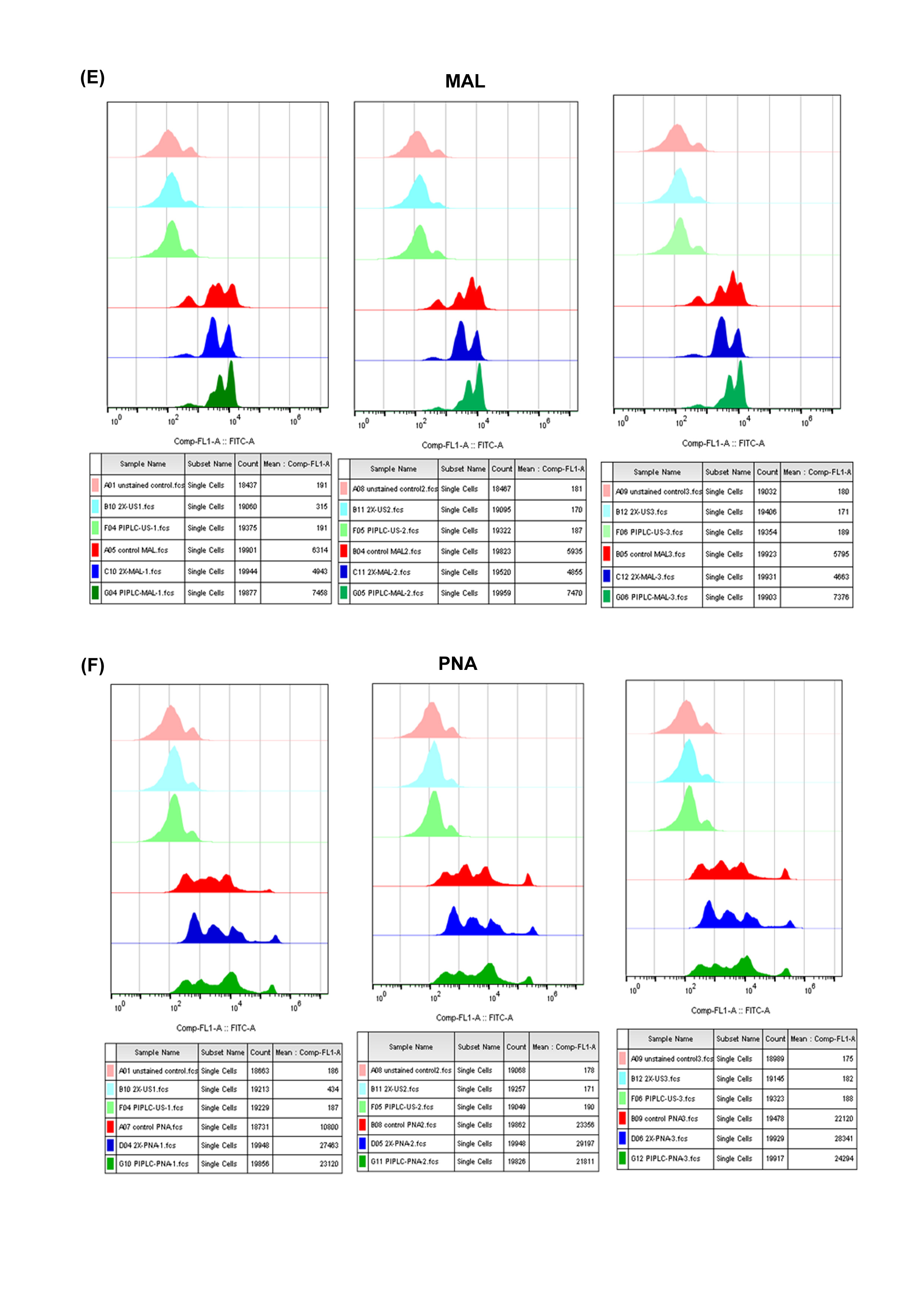

Supplement: Supplementary file 3 — Additional file 3: Supplementary Figure 3. The removal of glycans after salt and PI-PLC treatment. Overlay of the MFI histograms obtained by flow cytometry analysis of control, elevated salt and PI-PLC treated spermatozoa from buffalo bulls (n = 3 incubated with six FITC-labelled lectins viz. ABL (A) JAC (B) LEL (C), LCA(D), MAL-II(E) AND PNA(F). [file 12864_2021_7640_MOESM3_ESM.zip › Supplementary Fig. 3-3 .jpg]

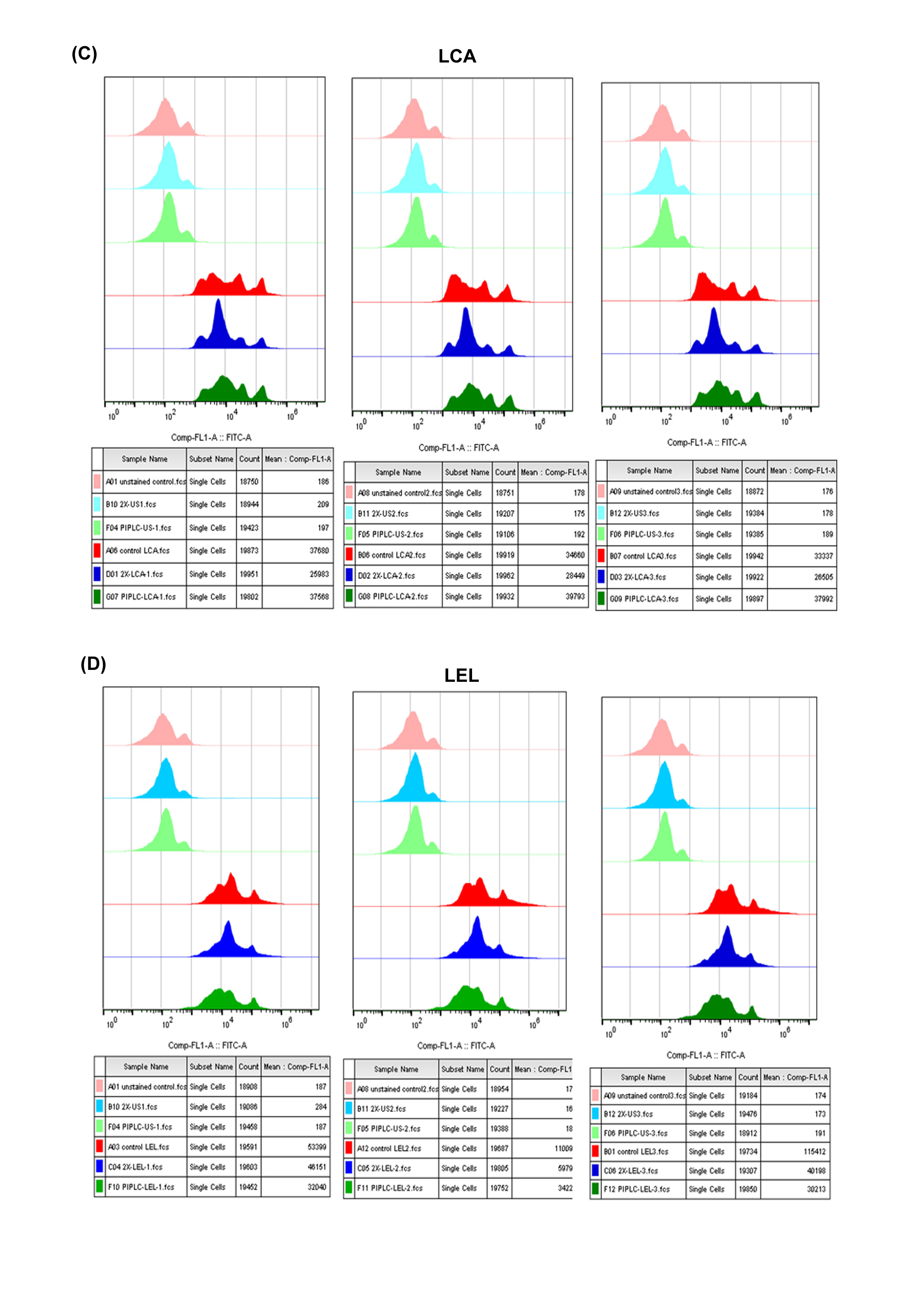

Supplement: Supplementary file 3 — Additional file 3: Supplementary Figure 3. The removal of glycans after salt and PI-PLC treatment. Overlay of the MFI histograms obtained by flow cytometry analysis of control, elevated salt and PI-PLC treated spermatozoa from buffalo bulls (n = 3 incubated with six FITC-labelled lectins viz. ABL (A) JAC (B) LEL (C), LCA(D), MAL-II(E) AND PNA(F). [file 12864_2021_7640_MOESM3_ESM.zip › Supplementary Fig. 3-2.jpg]

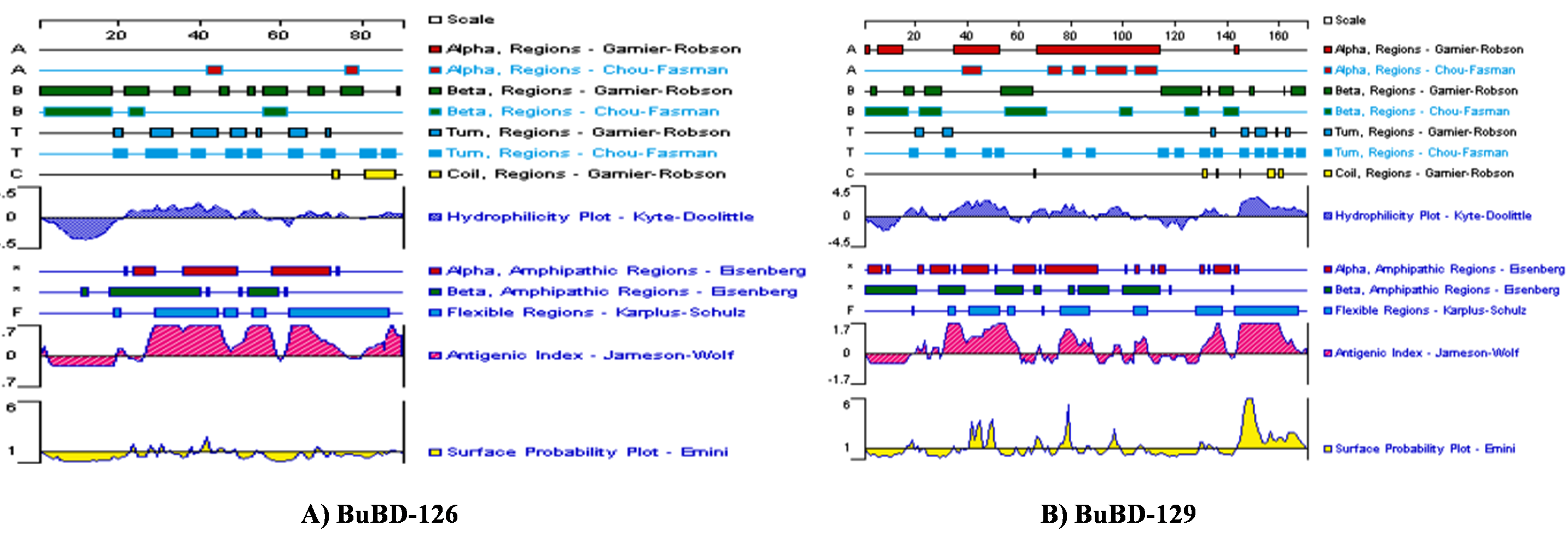

Supplement: Supplementary file 4 — Additional file 4: Supplementary Figure 4. Surface epitopes make better antigens. B-epitope mapping for BuBD-126 (A) and 129 (B) illustrating the results from IEDB server’s collection of tools and methods [file 12864_2021_7640_MOESM4_ESM.tif]

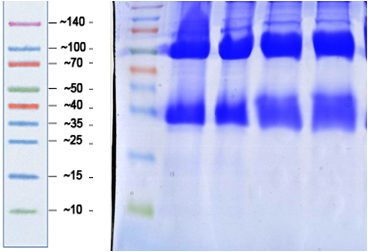

Supplement: Supplementary file 5 — Additional file 5: Supplementary Figure 5. The purified IgGs from rabbit serum. Reducing SDS PAGE of the affinity-purified IgGs depicting the major bands near 25 kDa and 50 kDa, corresponding to the light chain (L) and heavy chain (H) of the IgG antibody. [file 12864_2021_7640_MOESM5_ESM.tif]

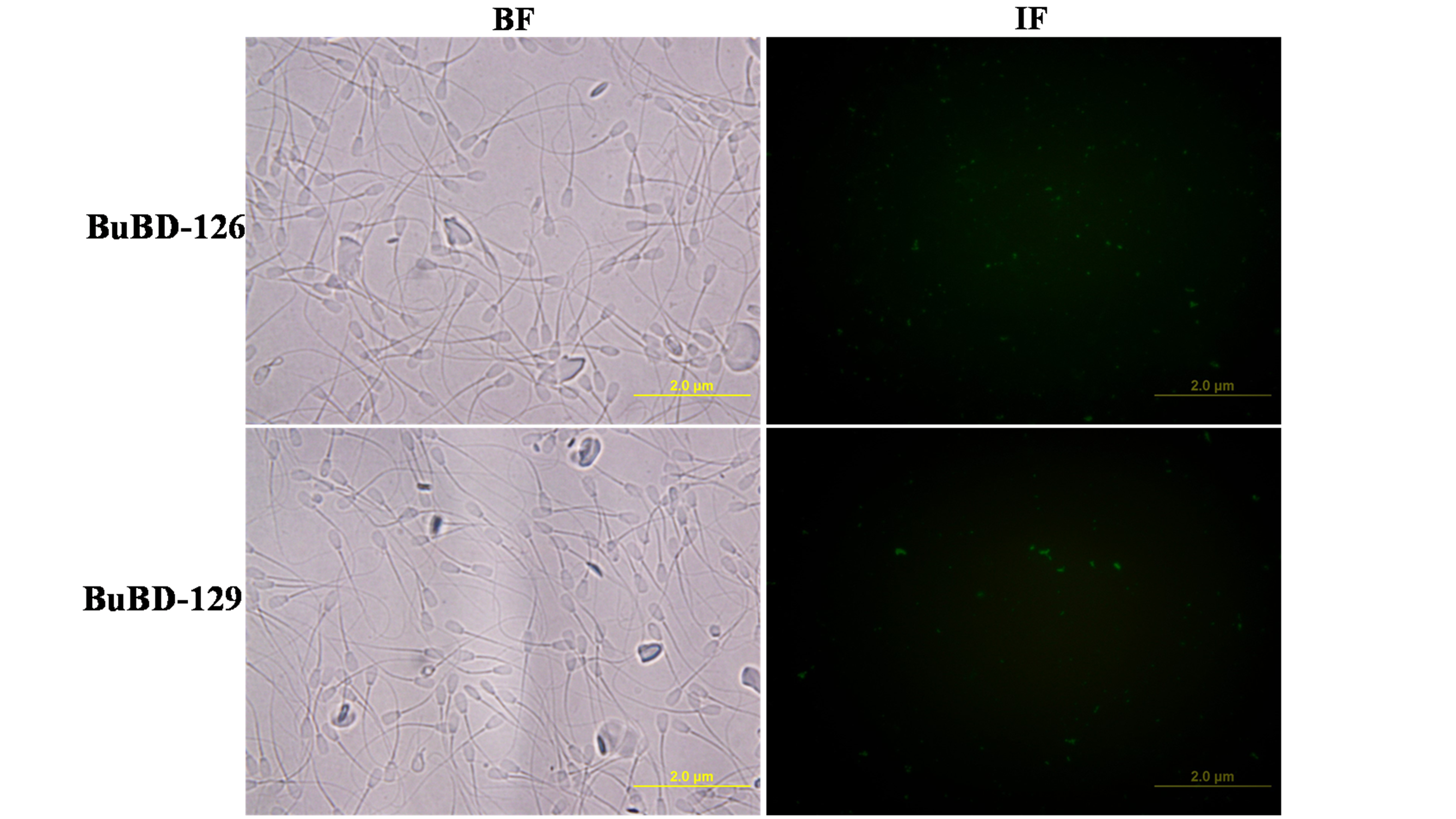

Supplement: Supplementary file 6 — Additional file 6: Supplementary Figure 6. Negative controls for BuBD-129 and 126. Bright field and fluorescent micrographs of the negative controls for the anti-BuBD-126 and 129 primary antibodies. [file 12864_2021_7640_MOESM6_ESM.tif]
